# Staying positive: producing net power

**DOI:** 10.1098/rsta.2023.0404

**Published:** 2024-10-09

**Authors:** Jack Acres, Ioannis Antoniou, Finlay Christie, Daniel Blackburn, Samuel Knight

**Affiliations:** ^1^ UKAEA (United Kingdom Atomic Energy Authority), Culham Campus, Abingdon, Oxfordshire OX14 3DB, UK

**Keywords:** STEP, power, energy, fusion, powerplant, thermodynamics

## Abstract

The Spherical Tokamak for Energy Production (STEP) prototype powerplant (SPP) will be a first-of-a-kind powerplant—its prime objective is to export electrical power, to the national power transmission system (‘grid’), above 100 MWe. As part of a wider issue, addressing the STEP concept design, this article seeks to explore how electrical power will be generated from a spherical tokamak heat source. Accordingly, the following key functions of the SPP power infrastructure are reviewed.

*Cooling the tokamak*: cooling the tokamak while extracting useful thermal energy.

*Generating power*: conversion of thermal energy to electrical energy (power generation).

*Managing energy*: management of the site-wide distribution, storage and energy export.

In each of these areas, the design scope, challenges and solution spaces have been discussed. This has shaped the design of the SPP power infrastructure, which in turn has ensured a powerplant design focused on operability and performance. Furthermore, it has been demonstrated that the SPP will achieve its prime objective in generating net power, which is enabled by a unique power infrastructure. Confidence in the ability to generate net power will be refined as the design matures. Finally, this article recommends key opportunities that STEP could use to improve power generation and reduce the parasitic load of the SPP.

This article is part of the theme issue ‘Delivering Fusion Energy – The Spherical Tokamak for Energy Production (STEP)’.

## 1. Introduction

**Figure 1.**
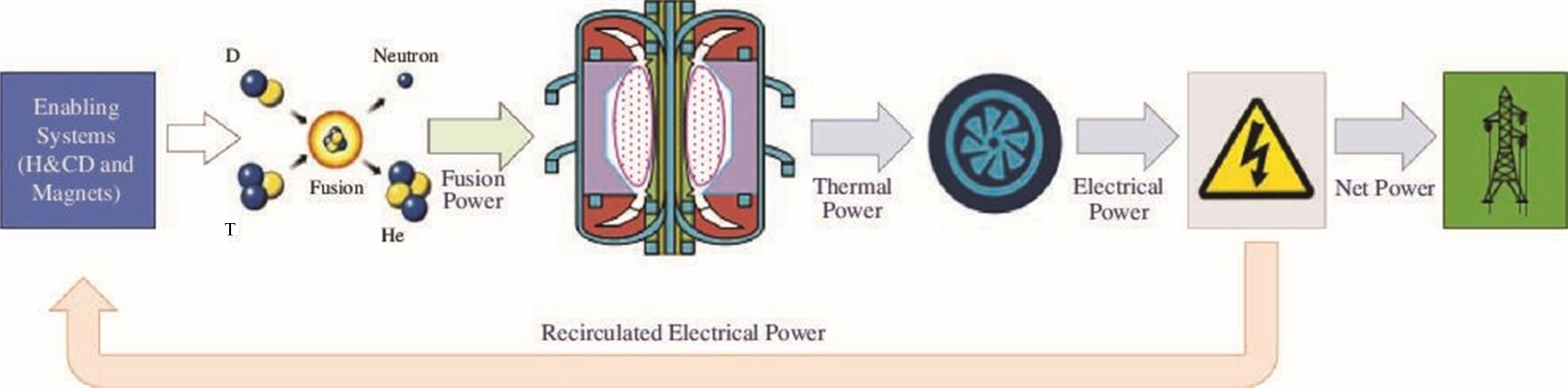
The SPP net power generation flowsheet.

Conversion of fusion heat into electrical power is the current paradigm of many fusion projects. First, the neutron and radiative heat resulting from the fusion reaction must be removed through multiple coolants within the tokamak. This process uses dedicated coolants for each in-vessel component (IVC). The heat from the IVCs must be integrated into a thermodynamic cycle, which in turn will generate electrical power. This power must then be managed across the plant, as tokamaks have notoriously high and unique power demands (in the context of modern powerplants). Magnets and the heating and current drive (HCD) systems are some of the most significant power users. The power transfer and conversion paths are summarised in figure 1.

The Spherical Tokamak for Energy Production (STEP) power infrastructure team is responsible for the design of the thermal and electrical energy management across the SPP; it is also responsible for the thermal-to-electric conversion. The SPP power infrastructure is split into multiple systems to achieve its functions:

— *Power cycle and cooling system*, which contains the following subsystem:
  - *Thermal power transfer system (TPTS)*: this system integrates the coolant and heat from the IVCs and transfers the heat to the thermodynamic cycle.
  - *Working fluid and power generation system (WFPGS)*: this system uses the integrated heat within a thermodynamic power cycle to generate power.
  - *Site water and waste heat system (SWWHS)*: this system rejects the low-grade waste heat from the power cycle and parasitic loads to the environment using cooling systems.
  - *Cryogenics system*: this system will meet the requirements of several subsystem that require cryogenic refrigeration, for example, superconducting magnets.
— *Electrical infrastructure*, which contains the following subsystem:
  - *Electrical distribution network (EDN)*: this system is responsible for the distribution of electrical power to different areas of the SPP.
  - *Grid connection system (GCS)*: this system forms an interface between the SPP and the grid.
  - *Central energy storage system (CESS)*: this system stores energy that is dispatched during the start-up of the SPP.

We discuss the contextual background and design scope of the power infrastructure’s primary functions: cooling the tokamak, generating power and managing energy (N.B. certain functions are met by multiple systems). We then focus on the challenges and technological solution space for the various systems. Critical design trades made, which will ensure an efficient, flexible and safe integrated solution, will be discussed. In pertinent areas, the importance of commercial viability will also be outlined. Finally, we review the holistic challenges of achieving net positive power from the STEP heat source, during this conceptual design stage of the SPP.

## 2. Cooling the tokamak

### (a) Heat generation from fusion

The SPP relies on the fusion of deuterium and tritium nuclei. This fusion reaction releases large amounts of power (known as fusion power or *P*_fus_) as both neutronic heating and alpha particle heating. The heat that must be removed from the tokamak is not solely from fusion power. Tokamaks use the HCD system to sustain the plasma. This also adds power into the plasma, referred to herein as *P*_aux._ Within a powerplant setting, tokamaks must breed tritium as fuel. This is bred in the blanket using a mix of lithium isotopes and a multiplier; breeding and neutron multiplication is typically exothermic and generates heat in addition to the neutronic heat deposited in the blanket. The heat generated from breeding and multiplication is additional to the heat from fusion and must be considered as part of the total tokamak thermal heat output during a steady-state breeding operating scenario (which applies to both spherical and conventional tokamaks [1]). The heat released from breeding is referred to herein as *P*_breeding_. Heat developed from the neutron interaction with other materials in the vessel components (e.g. structural steels) is also accounted for in th*e P*_breeding_ term. Therefore, the total thermal power from the tokamak can be defined as


(2.1) $$P_{therm}=P_{fus}+P_{aux}+P_{breeding}.$$

For the SPP, a fusion power (*P*_fus_) of 1750 MWth is defined based on a range for the achievable plasma [2], the combined heat from breeding (*P*_breeding_) and the HCD heating (*P*_aux_) is 417 MWth (based on heat injected into the plasma and neutronics analysis), resulting in a total tokamak thermal power (*P*_therm_) of 2167 MWth.

To maximize power production, all heat from the tokamak should be captured and integrated into a thermodynamic power cycle, as far as reasonably practicable. The total *P*_therm_ heat developed within a tokamak can be categorized into different ‘types’ of heat:

— *Neutronic heating*: this will be a ‘volumetric heat load’ within the whole tokamak.
— *Radiative heating*: this is the radiation from the plasma, which will effect plasma-facing components (PFCs). This should be considered as a ‘surface heat load’ on PFCs.
— *Charged particle*: this is heat imparted from plasma inside the vessel through direct contact with certain PFCs. This should be considered as a ‘surface heat load’ on PFCs (primarily divertor PFC).
— *Breeding*: this is the heat generated by nuclear reactions within the blanket (including breeding reactions). This will be a ‘volumetric heat load’ within the blanket.

Understanding volumetric and surface heat loads is a key factor in defining the heat splits across the IVCs.

### (b) Design challenges and solution space

#### (i) Maximizing tokamak heat usage

**Figure 2.**
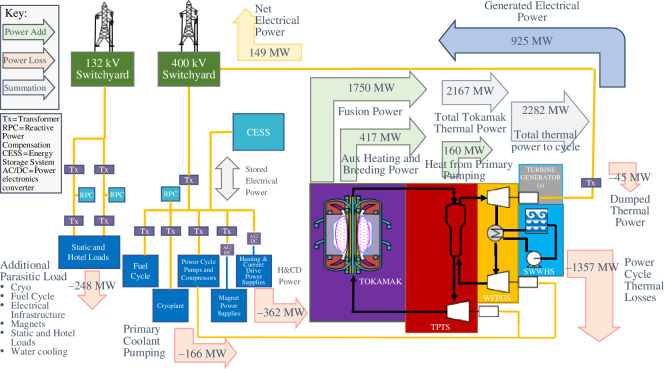
Power infrastructure conceptual architecture, showing net power flow.

Achieving net power is dependent on how much heat, generated within the tokamak’s IVCs, can be used to generate electrical power rather than rejected to the environment. Each IVC has specific, and typically unique, cooling requirements [3]. While it is recognized that maximizing heat usage for power generation is important, there are instances where the heat from an IVC coolant cannot be used. If the temperature of the coolant is very low (*ca.* 150°C or less), it is impractical to integrate this heat into the thermodynamic cycle. This can be because there is already a significant amount of low-grade heat (200–250°C) from other tokamak IVCs that displaces the ability to efficiently integrate further heat below this grade. This is the case for the inboard shield (inner) and the vacuum vessel for the SPP, where the coolant temperatures are too low and, therefore, the heat from these components will be rejected to the environment (or if possible, used for utility heating); as seen in figure 2 where a small portion (45 MWth) of the heat is ‘dumped’, hence heat from the tokamak which may be integrated for power generation is approximately 2122 MWth.

**Figure 3.**
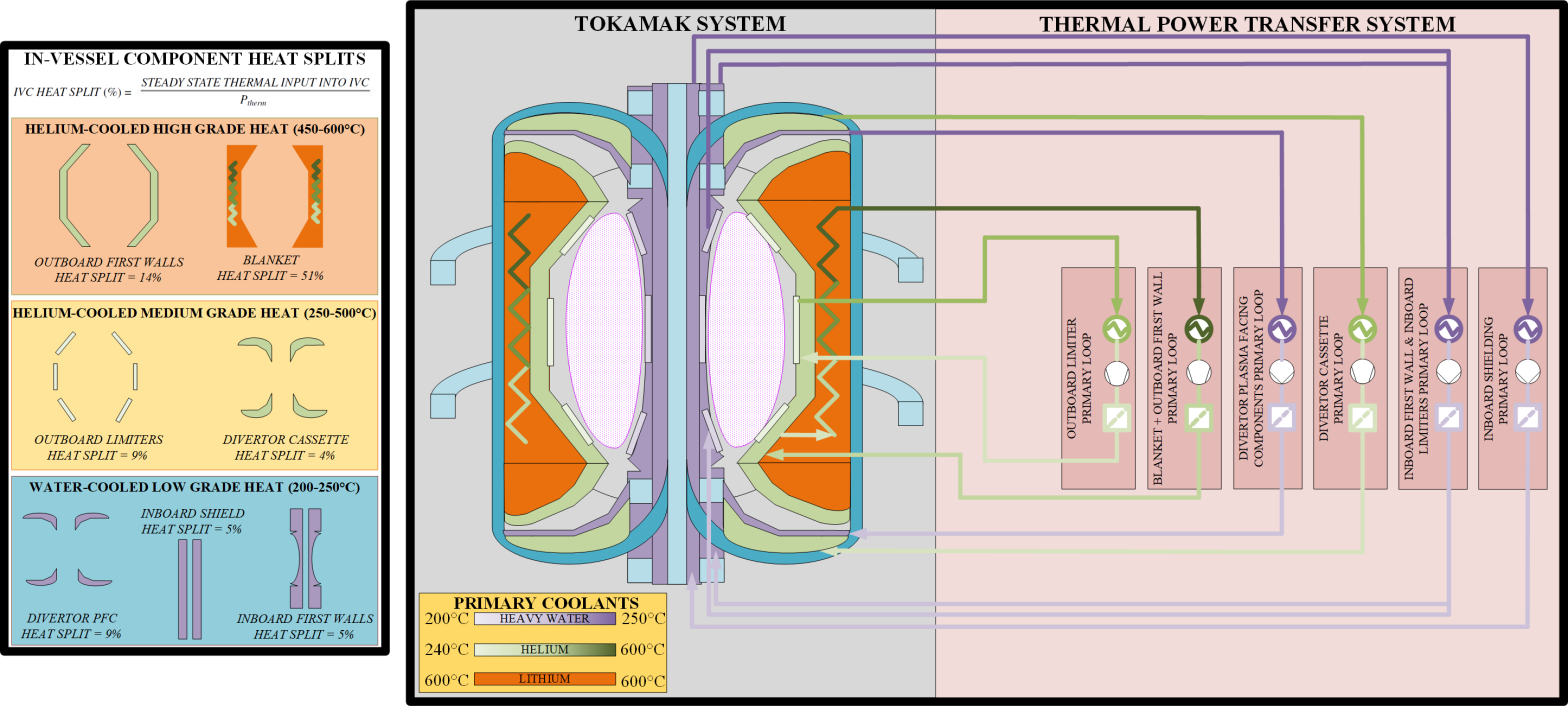
Overview of the primary coolant and Tokamak components heat splits.

Conversely, the heat from the blanket, first walls (inboard and outboard), inboard shield (outer), limiters and divertors will all be used for power generation as shown in figure 3. The combined heat extracted from these components make up 98% of the total tokamak thermal power (*P*_therm_). However, the integration of these heats into the thermodynamic cycle adds coolant parameter constraints for the IVCs in question (e.g. temperatures, discussed in §3).

#### (ii) Choosing tokamak coolants

Coolant selection is important to many areas of the plant design, IVC design, power integration and tritium breeding. The SPP IVC coolant choices are summarised in figure 3. A number of critical design trades were made, as part of the coolant selection for the IVCs:

— A liquid metal lithium breeder is necessary to achieve the desired tritium breeding ratio (TBR). This causes inherent complexity in design and adds safety risks [4].
— Water will be used for inboard components for moderation purposes, maximizing magnet lifetime and limiting cryogenic loads. Water temperatures will be limited by component allowable operating pressures (as phase changes must be avoided), which in turn can limit heat integration into the power cycle and have a negative effect on net power. There is also a safety consideration when water and liquid metals are used in the same tokamak machine.
— Water was selected to cool the high heat flux divertor PFC as it ensures a sufficient heat transfer coefficient. There are water temperature constraints, as discussed in the previous point. The TBR can be marginally improved by using ‘heavy water’ (D_2_O) instead of water. Molten salts and helium were de-selected owing to poor heat flux handling. The use of lithium was not advised owing to safety and technology readiness level. Both molten salts and lithium pose a significant loss of cooling accident risk.
— The outboard first wall and blanket coolant need to ensure neutron transparency (for improved tritium breeding), be safe to use with the liquid lithium breeder, minimize corrosion problems with structural materials and minimize activation products. Helium was therefore selected. This accepts the negative effect of net power owing to the large parasitic load required for coolant circulation.
— The number of coolants is limited to ensure that maintenance is viable. More than two coolants in the machine incur machine and plant complexity, hindering design, installation and maintenance [5].
— There are no direct interfaces between reactive coolants (i.e. water and lithium).

#### (iii) Managing multiple coolants

The TPTS is responsible for integrating multiple IVC primary coolant loops with the thermodynamic cycle. Among other contraints, the TPTS must ensure:

— *Coolant circulation (pumps and compressors)*: helium compressors at the SPP’s conditions will require significant technology development.
— *Coolant purification*: the coolants will be exposed to high neutron fluxes and as a result are expected to develop harmful impurities that can reduce component lifetimes and impose safety risks; therefore, effective and on-going chemistry control is required. Moreover, coolants will become tritiated after exposure to the IVCs and must be de-tritiated [4].
— *Coolant inventory management*: owing to operational regimes of the SPP, routine filling and draining of coolants is required, and hence requires on-site storage and disposal routes.

#### (iv) Minimizing pumping powers

Gas coolants will incur large parasitic loads, through gas compression. The TPTS will minimize this by considering:

— *Maximum coolant pressure*: ensuring a high-pressure gas coolant loop equates to a lower compression ratio, minimizing compressor power demands. However, high pressures may limit the design of the IVC, for the blanket this can restrict the TBR (owing to larger structural volume).
— *Heat-exchanger design*: adapting the heat-exchanger design to minimize this pressure loss will be critical in reducing pumping powers. This will result in a trade between sensible heat-exchanger designs and optimising for pressure loss, at a given heat-exchanger efficiency.
— *Loop pressure losses and layout*: pressure losses across the loop overall must be minimized by maximizing pipe sizes and minimizing losses through manifolding and control elements. This can result in spatial design trades within allocated port spaces and in areas close to the tokamak.

#### (v) Cryogenic cooling

Cryogenic refrigeration is required by several of the SPP’s subsystems, including the superconducting magnets, which require 20 K cryogens. The thermal shield and cryopumps also require refrigeration. Cryogenic refrigeration will be supplied by a cryoplant with an associated distribution system, using helium as the refrigerant. The total power consumption of the cryoplant is estimated to be 20–30 MWe. The cryoplant will satisfy four temperature points.

## 3. Generate power

### (a) Power generation methods

Once the heat has been generated and extracted from the tokamak, it is transferred to a power generation cycle for conversion to electricity. By far, the most common power cycle in powerplants today is the steam Rankine cycle (SRC), although other technologies exist such as variants of the Brayton cycle.

In a fusion context, primary coolants typically transfer the thermal energy gained into a separate working fluid (secondary coolant) through a heat exchanger. This working fluid is then used in a prime mover to create mechanical energy (e.g. a steam-driven turbine—where steam is the working fluid); this is an example of an indirect cycle configuration. Direct cycles, where the primary coolant and working fluid are the same, were discounted as a possibility in the design of the SPP. A direct cycle simplifies the design of the total powerplant. Conversely, a direct cycle either constrains the power cycle or the IVC primary coolant conditions, resulting in an excessively low power generation or an unrealistic IVC design, respectively.

The mechanical energy produced in the prime mover is converted into electrical power in a directly connected generator. The generator producing electrical power is synchronized to the grid. One of the challenges associated with the power generation system is sustaining the inherent flexibility to withstand prototypic activities, while also ensuring high efficiencies to deliver net power.

### (b) Thermodynamic power cycles landscape

**Figure 4.**
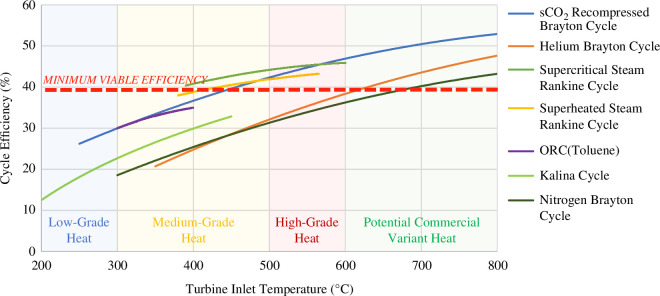
Efficiencies versus temperatures of state-of-the-art thermodynamic cycles.

During the early stages of the SPP design, many thermodynamic cycle technologies and combinations thereof, were considered for their applicability as the SPP’s power cycle. The performances of these cycles are summarized in figure 4. This figure also shows a minimum viable efficiency threshold. This threshold is calculated by considering the thermal power, that can be integrated into a power cycle from both the tokamak (approx. 2122 MWth) and the primary pumping (approx. 160 MWth). The total added thermal power into the power cycle (approx. 2282 MWth) and the total plant parasitic loads (approx. 776 MWe), as per figure 2, are used to calculate a minimum viable efficiency of approximately 39% needed for 100 MWe net power export to the grid.

This minimum viable efficiency assumes that the majority (98%, as defined in §2) of the heat from the tokamak may be integrated into (ideally) one thermodynamic cycle or multiple cycles. For a single thermodynamic cycle, the turbine inlet temperature is driven by the blanket coolant temperature (or high-temperature heat) and it is assumed the heat from other IVCs is integrated at various stages of the cycle (e.g. water pre-heat for SRC). Designs which use multiple cycles will have different turbine inlet temperatures driven by different IVC coolant outlet temperatures.

A summary of the possible cycles and cycle configurations for the SPP is outlined below:

— *Superheated SRC* is the paradigm for most fusion powerplants [6]. It is a low-risk, established technology that could potentially be adapted to suit the needs of a fusion powerplant. The key challenges associated with this cycle (for the SPP) include risk of large heat rejection owing to heat integration challenges and low flexibility when coupled with a highly dynamic fusion application. Supercritical steam cycles can also be envisioned, in this instance, a trade must be made to adapt a less established technology for slightly higher efficiencies at a given temperature.
— *Supercritical carbon dioxide (sCO*_*2*_ *) Brayton cycle* and its variants present an opportunity for greater heat integration, while being better suited to the flexibility demands of prototypic operation. However, this technology is currently immature, with no demonstration at the required scale. The sCO_2_ technology is inherently more compact which allows for more adaptability versus dynamic scenarios, it will also be cost effective (after first development) and will occupy less footprint [7].
— *Helium and nitrogen Brayton cycles* will not meet the minimum required efficiencies at the temperatures of interest for the SPP and are therefore not suitable.
— *Organic Rankine cycle (ORC*) *and Kalina Rankine cycle* (two-fluid mixture, such as water or ammonia) are currently used for smaller-scale applications. It has been considered for the SPP in combination with other cycles to recover the low-grade waste heat; however, evaluation of this combined cycles technology has shown incompatibilities regarding safety, size and complexity aspects of the whole plant. For these reasons, these technologies are not suitable for the SPP.

There are therefore only two viable options to ensure minimum viable thermodynamic efficiencies with the available heat from the tokamak: sCO_2_ Brayton cycle (a recompressed Brayton cycle variant) and SRC (superheated or supercritical).

### (c) Design challenges and solution space

#### (i) Achieving temperatures

To increase power output from the SPP, temperatures from the IVCs must be maximized. The efficiency of the power cycle is directly controlled by the blanket outlet temperature, as this is both the highest temperature achievable and the largest amount of heat. Nonetheless owing to the heat splits and the large amount of heat deposited into the other IVCs, the temperatures of these other IVCs will have an effect on the ability to optimize the power cycle—ultimately affecting the thermal-to-electric conversion.

It is important to maximize the temperature for the prototype demonstration, but this is also a key factor for commercial viability. Furthermore, temperatures may be demonstrated for other high-temperature industrial processes, supporting a wider global decarbonisation beyond the grid [8].

#### (ii) Integrating heat and ensuring efficiency

Heat recuperation and integration within the cycle are critical when maximizing efficiencies. Heat which is linked to individual IVCs of the tokamak and operating at different conditions, must be integrated into the thermodynamic cycle(s). A cycle that can add versatility and margin to heat integration will be better placed to incorporate as much of the heat from the tokamak (*P*_therm_) as possible, improving efficiencies and net power output. This has been an important factor in the power cycle development for the SPP. Heat integration has been maximized for both the SRC and sCO_2_ Brayton cycle technologies (within a single thermodynamic cycle) resulting in comparable thermal-to-electric-conversion efficiencies from a single tokamak design point.

The heats from the different IVCs, which are extracted with coolants operating at varying temperature and pressure conditions, must be integrated sequentially and between turbomachinery components and stages. Heat must also be integrated between heat recuperative steps within a thermodynamic cycle, this is especially true for the sCO_2_ Brayton cycle technologies [9].

Other design parameters may also be considered to maximize the overall cycle efficiency such as:

— *Cycle layout and location*: in proximity to the tokamak and component location relative to each other.
— *Component design*: heat-exchanger and turbomachinery efficiencies.
— *Energy losses from the system*: heat and pressure losses from components/piping.
— *Heat rejection to the environment*: considering seasonal effects and cooling system efficiency.

#### (iii) Ensuring flexibility

**Figure 5.**
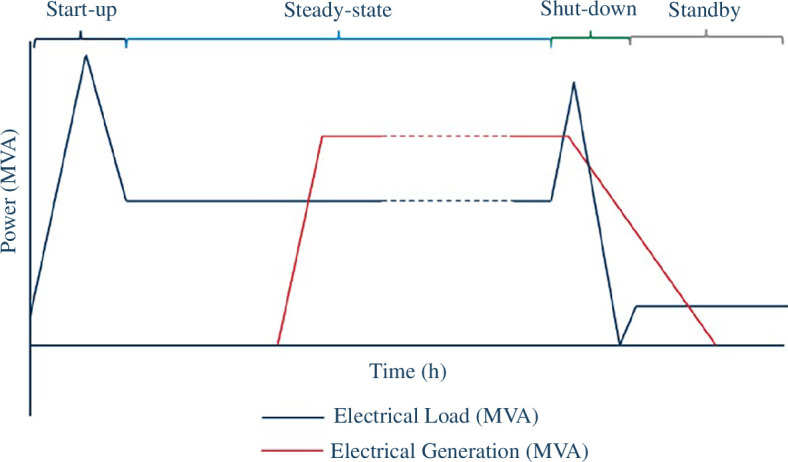
The SPP power profile.

Operational flexibility will be required during all phases of STEP prototypic operations. This is emphasized when considering the dynamics of plasma and pulsed operations of the SPP (as shown in figure 5), translating into rapid and frequent start-up/shut-down regimes for the SPP relative to conventional powerplants.

To support this dynamic requirement, the WFPGS will provide the following functions:

— *Availability*: ensuring that the WFPGS is available without fusion power and ready to receive fusion power.
— *Independence*: ensuring the WFPGS equipment can be controlled independently, allowing a safe shut-down of the systems during a trip and increasing chances of a recovery after a plasma disruption.
— *Variation handling*: ensuring consistent powerplant performance during unknown operational regimes and plasma performance. This will ensure predictable external interface requirements.

Auxiliary heat, which can be used to run the power cycle independently of tokamak operations, will ensure the WFPGS can achieve these functions. Auxiliary heat may be provided from energy storage solutions or an alternative heat source—both have benefits and drawbacks [10]. As commercial viability is demonstrated, this need is diminished owing to operational predictability.

Thermodynamic cycles which can accommodate auxiliary heat and supplement it are an important factor in the WFPGS solution development. This will include how inherently adaptable and flexible a cycle is in these dynamic scenarios. The sCO_2_ Brayton cycle technology and its compact turbomachinery, lends itself well to these scenarios.

#### (iv) Whole-plant power cycle solution space

A power cycle solution is required to address the fusion-specific challenges of the SPP. The sCO_2_ Brayton cycle technology presents an interesting opportunity to maximize both efficiency and flexibility around the SPP operations—and therefore is being pursued for the SPP. Nonetheless, the development risks linked to sCO_2_ Brayton cycle technology are acknowledged, hence the SRC technology remains a fallback position and is studied in conjunction. Using figure 4, for a sCO_2_ Brayton cycle with a turbine inlet temperature close to 600°C (driven by the blanket coolant outlet temperature defined in figure 3), a thermodynamic efficiency of approximately 45% could be estimated for the SPP design point. However, some conservatism must be applied to account for the added inefficiencies and energy losses (pressure and heat) linked to complex heat integration, especially owing to the temperature loss across the primary blanket heat exchanger, affecting the maximum turbine inlet temperature. For these reasons, an efficiency of 40–41% is estimated, resulting in a 925 MWe output as per figure 2.

## 4. Manage energy

### (a) Overview of the SPP electrical power demand

Initiating and sustaining nuclear fusion reactions requires a significant energy input. In a tokamak, the plasma particles must reach extreme temperatures, up to hundreds of millions of degrees Kelvin. On the SPP, owing to the ‘non-inductive’ flat-top, which allows for continuous non-pulsed operation ([11]), plasma current is induced exclusively with external radio-frequency heating sources. The HCD system transfers high-frequency electromagnetic radiation to the plasma via radio frequency (RF) heating [11]. Existing HCD technologies remain relatively inefficient (40–45%), which results in significant parasitic losses throughout the SPP operation (approx. 362 MWe during steady-state, figure 2). The primary source of this inefficiency stems from the conversion of electrical power to RF power (gyrotron efficiency).

In addition, tokamaks use strong magnetic fields to initiate and control the plasma, this is another major source of power consumption. The SPP will use superconducting magnets to confine the plasma, which are deemed crucial owing to the very high electrical currents needed to confine the plasma [12]. This necessitates a large cryoplant that can ensure the superconducting magnets are operated at suitably low temperatures. The electrical power demand of these systems is combined with other users including: the fuel cycle, water cooling and building loads, to give an ‘additional parasitic load’ of approximately 248 MWe, as shown in figure 2.

Pumping of the primary coolants (as defined in figure 3) will require a significant electrical supply (approx. 166 MWe), this is linked to the gas coolant requirements as defined in §2. In addition, the compact nature of the spherical tokamak and its IVCs will incur added pressure losses leading to high compression power requirements. The majority of this power (160 MWth) will result in heating of the coolant, owing to assumed motor efficiencies of approximately 96%.

The thermal energy produced within the tokamak is to be extracted and coupled with a thermodynamic power cycle technology (see §3), aiming to export power to the grid. Losses linked to pumping of the working fluid and other inefficiencies in the cycle are accounted for in the thermodynamic cycle efficiency.

Future technology improvements could enhance the efficiency of the plasma HCD and thus reduce the total electrical losses of the system, but they will remain a challenge, especially during the start-up and shut-down of the plasma. A selection of the SPP’s steady-state electrical loads are highlighted in figure 2. The power cycle and plasma heating loads represent the bulk of overall electrical losses.

### (b) Design challenges and solution space

#### (i) Operations and dynamics of the SPP

A simplified view of the operation of the SPP is provided in figure 5. The start-up period describes the state in which the plasma is ramped up and in which the SPP’s parasitic loads are expected to reach their peak value. The SPP’s electrical power demand outside of steady-state will need to be sourced independently of the SPP’s power generation and will be hundreds of MWe. The current concept design assumes that a combination of power import from the grid (at the 400 kV transmission network connection node) and power from a CESS, which is embedded within the EDN, could cater for those high parasitic loads. The exact split between the two power sources will be dependent on the electrical characteristics of the 400 kV grid connection at the West Burton, Nottinghamshire, site. The majority of the SPP’s fast power transients are expected to be supported by the CESS, to minimize the effect on the grid connection.

Once steady-state/flat-top operation is achieved the fusion power then can be used to drive the power cycle and thus the SPP could start producing its own electric power. This process would not be instantaneous as the power cycle will possess its own time constants and thus electric power production would lag. The time that it takes for the SPP to output its full electric power, and thus supply both its internal electrical needs as well as export net power to the grid, would determine the requirements for electrical energy that would need to be sourced from the GCS and the CESS.

Procuring such large amounts of electric power and energy (as described in figure 2) to initiate and sustain the fusion reaction is a significant challenge for any electrical infrastructure system that is required to support a fusion powerplant. At the same time, maintaining voltage levels within acceptable limits across the electrical infrastructure adds more complexity to this challenge, requiring adequate reactive power compensation mechanisms to be deployed. Establishing a strong electrical connection with the grid could reduce this challenge, but for future commercial applications where strong grid connections may not be available, there will probably be a strong dependency on the CESS to support both active and reactive power requirements.

#### (ii) SPP electrical distribution and power source integration

A high-level overview of the SPP’s electrical infrastructure is shown in figure 2. The EDN will distribute electrical power to all of the plants electrical loads according to their nominal ratings. It will comprise of an array of protection, control and conditioning (reactive power compensation and harmonic filtering) equipment which will ensure that all site areas receive adequate levels and quality of power in a safe and reliable way. The current concept design splits the SPP’s loads into two, with the steady-state loads, mainly non-fusion loads, connected to a 132 kV distribution network node (steady-state EDN) and the pulsed loads connected to the 400 kV transmission network (pulsed EDN). This approach ensures that the constant power loads are decoupled from the power transients linked with the fusion loads.

**Figure 6.**
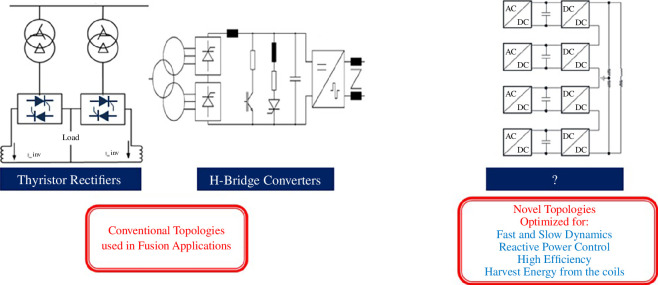
Power converter technologies for fusion powerplants.

The interconnection of three different power sources (CESS, grid, power generation) through the SPP’s pulsed EDN is another significant challenge. Each power source will have a unique operational profile and dynamic characteristics. Hence, the integrated control of the three power sources must achieve a balanced overall power and energy flow across all operational phases. Handling different load dynamics and responding to off-normal events would add complexity into this control task [13]. In addition, the current concept design assumes a single point of connection for power import and export at the 400 kV transmission network connection node. A design deploying local electrical energy storage (independent to the CESS), or load-shedding equipment closer to the systems that exhibit fast dynamics, could provide isolation from their transient effects and ensure that the power generation does not lose synchronism with the grid. Some of the fastest dynamics in the SPP are related to controlling the magnets and the HCD output. These systems are connected to the pulsed EDN through a series of power converters, each with different technical requirements ranging from high voltage and low current to high current and low voltage. These power converters will be the backbone of the SPP fusion powerplant and must have very high efficiency (to minimize overall parasitic losses) in all operating regimes, as well as exhibit very high reliability. Power converter topologies (figure 6), inspired by the renewable energy industry, could enhance the control of plasma current and improve plant operations during transient events.

## 5. Staying positive: net power holistic evaluation

### (a) Net power definition

Net power is defined as the net electrical power produced during steady-state flat-top Deuterium - Tritium (D-T) operations after accounting for all parasitic loads required for operations. The SPP aims to demonstrate a 100 MWe minimum export to the grid. As a prototype, the following conditions apply to the SPP when demonstrating net power:

— Only electrical power converted from thermal power (*P*_therm_) from the tokamak (and heat from primary pumping) will be used to count towards net power generation and to meet all parasitic loads.
— Net power demonstration at flat-top does not require net energy generation over the course of a whole experimental pulse or any other time period (i.e. net power pulses need not account for additional energy required for start-up and shut-down, or to sustain system between pulses).
— Net power operation must be repeatable and stable.

It is expected that a commercial plant will run for order of magnitude longer periods, making some of these considerations irrelevant.

### (b) Net power evaluation and design effects

Net power to the grid is evaluated using the net-power equation:


(5.1) Netpowerexporttothegrid=Electricalpowergenerated–Internalparasiticloads

Figure 2 illustrates how the different elements of this equation sit within the conceptual architecture of the SPP power infrastructure. The blue arrow represents electrical power generated (925 MWe), the red arrows represent internal electrical parasitic loads (776 MWe in total) and the yellow arrow represents net power exported to the grid (149 MWe). These figures represent a best-guess scenario, around which there remains uncertainty stemming from performance; uncertainty of both plasma and individual technologies.

As per equation (5.1) and figure 2, to maximize net power and improve confidence, one should focus on:

— The ability to maximize power generation (see §2 and §3).
— The ability to manage energy as efficiently as possible, minimizing parasitic loads and distribution losses across the powerplant (see §4).

Net power is not the only consideration of the SPP and many design trades are necessary to achieve a feasible, holistic plant design. Key examples of design trades have been discussed in this article (i.e. coolant selection, coolant temperature, spatial constraints and direct versus indirect power cycle). Further design trades include:

— *Fusion power:* higher fusion powers can increase net power but will increase size and costs of the powerplant.
— *HCD requirements for the plasma:* plasma scenarios that require the least HCD input (high-confinement mode operation) are also more susceptible to edge limiting mode disruptions, creating a trade-off between the likelihood and simplicity of achieving stable and safe power-producing plasmas, and the net power they may produce [11].
— *PFC heat fluxes:* designing a more compact plant may reduce capital expenditure but increases heat fluxes on the PFCs thereby increasing coolant pumping powers [3].
— *Maintainability:* remountable joints in the toroidal field coils improve the ability to carry out plant maintenance and reduces its cost. However, coil joints introduce resistive losses in the magnets, increasing their power requirement and increasing the size of the required cryoplant [12].

### (c) Net power holistic challenges

#### (i) Driving efficiencies across multiple concepts

The power balance model (PBM) software has been developed by the STEP Power Infrastructure team to analyse the power balance of a fusion powerplant. The model estimates the electrical power consumption the SPP’s different systems and the plant’s power generation using a suite of bespoke models that are inter-linked with a Python Application Programming Interface [14]. The PBM was used extensively throughout early stages of the SPP concept design as it enabled an assessment of many configurations and their ability to export power. It was also used to validate design decisions that could improve the efficiencies of various systems within the SPP and hence would improve confidence in net power export. As the SPP concept has evolved, the evaluation of net power has matured. Power generation and coolant pumping powers are defined with high-fidelity process modelling. Individual parasitic loads are determined by system design following a single design point (with upper and lower predictions). The data captured is combined allowing an overall estimation of net power and its uncertainty to be made. The process helps drive efficiencies across the SPP design by integrating estimates from multiple teams, firmly embedding the net power target in the design process.

#### (ii) Reducing uncertainty and driving confidence

Confidence in net power is driven by uncertainty within the underlying parasitic load and power generation estimates. This is related to the maturity of the SPP concept design, as well as the inherent uncertainty in achievable plasma performance. As the SPP design evolves, so will the constituent systems, ensuring a better understanding of performance after the refinement of power generation and consumption data. Furthermore, as the SPP plasma scenario and tokamak structure develop, there will be an improved performance understanding, which will reduce uncertainty in the total power generation available during flat top.

#### (iii) Critical opportunities to increase net power

Part of the SPP’s mission is to provide a *path to the commercial viability of fusion*. To be cost-competitive, a commercial fusion plant will need to increase the overall efficiency with which fusion power is converted to exportable net electricity. This article has discussed key technology areas that affect net power, and it is of no surprise that many of these present the main critical areas of future opportunities:

— *Fusion power optimi*s*ation*: for a given design and a constrained geometry, fusion power should be optimized, capturing holistic impacts on net power, which may be counter-intuitive.
— *Power cycle efficiency*: this is critical in maximizing net power and is largely dependent on IVC temperatures. Research into new materials may present opportunities to increase the temperature of coolants—thereby unlocking new power cycle efficiencies and improving heat integration.
— *Pumping powers*: pumping of the primary coolants is a significant parasitic load. Altering and optimising the design around relaxed IVC requirements may provide the space for radical changes (such as coolant types), significantly reducing pumping powers.
— *Plasma performance and HCD efficiencies*: HCD is predicted to be the largest parasitic load for a STEP-like powerplant. To increase net power, plasma scenarios with low HCD input requirements using efficient HCD technologies, such as electron Bernstein waves, need to be realized. System efficiencies of the HCD systems have an impact but become less important as the required amount of HCD reduces [15].
— *Magnet and cryopump technology*: this is a significant parasitic load in the current SPP concept. Key opportunities lie in exploring the use of superconducting cables for distributing power to the magnets and optimising the cryoplant and cryo-distribution systems. Exploring even higher temperatures superconducting technology is also critical.

## 6. Conclusion

As part of the wider STEP concept design, the SPP power infrastructure design must ensure both plant operability and the achievement of STEP’s prime objective: the generation of net power. To this end, the following power infrastructure functions have been discussed:

— *Cooling the tokamak*: cooling of the tokamak while extracting useful thermal energy results in a complex design space where many integration trades must be made. The IVC coolant selection has been managed holistically to ensure the STEP objectives are met. Heat splits, temperatures and operating/configuration parameters have been shown to be important factors.
— *Generating power*: multiple thermodynamic technologies have been discussed with the merits and disbenefits illustrated. The sCO_2_ Brayton cycle is being investigated for its applicability to the SPP design and operation. However, it is recognized that there are development risks linked to the sCO_2_ technology, hence the SRC is still studied as a fallback option.
— *Managing energy*: the SPP’s electrical infrastructure manages energy across the plant. Significant parasitic loads, unique fusion power dynamics and the integration of multiple power sources have been shown to be key requirements. A robust design that incorporates a balance of electrical systems (e.g. energy storage) and a strong grid connection will be essential to support the SPP’s operations.

The current concept design point for the SPP shows that it will generate net power. The power infrastructure enabling this will also support the SPP’s operations at all stages. Moreover, net power holistic evaluations have identified opportunities to further drive efficiencies and improve confidence bands as the SPP design is matured. Overall, this remains an exciting design space for the first-of-a-kind SPP. An atypical approach to power generation and energy management is presented which will address the unique elements of the SPP and achieve its prime objective of delivering power to the grid. This has resulted in a novel concept design for the STEP power infrastructure which is based on a spherical tokamak heat source.

## Data Availability

Accessible data is shared via UKAEA Open Data Register to support the content of this paper [16].
